# Acute Carbon Monoxide Poisoning in a Filipino Household: A Case Report

**DOI:** 10.7759/cureus.50211

**Published:** 2023-12-09

**Authors:** Louraine J Bagares, Philip Rico P Mejia, Rene B Punsalan

**Affiliations:** 1 Department of Clinical Neurosciences, Section of Neurology, University of the East Ramon Magsaysay Memorial Medical Center, Quezon City, PHL; 2 Department of Neurology, Cardinal Santos Medical Center, San Juan, PHL; 3 Department of Neurology, University of the East Ramon Magsaysay Memorial Medical Center, Quezon City, PHL

**Keywords:** filipino, poisoning, acute carbon monoxide poisoning, filipino household, household, carbon monoxide, carbon monoxide poisoning

## Abstract

There are multiple reports already regarding acute carbon monoxide (CO) poisoning in the Philippines secondary to the misuse of portable generators, especially during times of typhoons. We present a case of unintentional carbon monoxide poisoning in a Filipino household wherein our index patient is among the five members who were unconscious before they were rushed to the hospital. Three of the household members, on the other hand, were found dead. The index patient had an increased serum fraction percentage of carboxyhemoglobin level and presented with rhabdomyolysis during admission. Neuroimaging confirmed a hypoxic-ischemic encephalopathy secondary to carbon monoxide intoxication. Even without hyperbaric oxygen therapy, the patient improved with adequate hydration, early rehabilitation, and trauma-focused psychotherapy.

## Introduction

The Philippines’ geographical location makes it highly susceptible to natural disasters, thereby producing numerous calamity-related damages and casualties [1]. In times of post-typhoon power shortages, diesel-powered portable generators have been used by many in the far-flung provinces [2]. However, the use of generators caused several accidental and unintentional carbon monoxide (CO) poisoning cases and deaths worldwide [3,4]. These pieces of equipment emit carbon monoxide and, when placed in an enclosed space, could cause a buildup of CO [5] and ultimately wreak havoc on patients.

We now present a case report of acute carbon monoxide intoxication in a Filipino household due to a portable generator. We specifically highlighted an index patient who was fully worked up during his admission.

## Case presentation

A 34-year-old male was brought to the emergency room due to altered mental status. He was seen by neighbors lying unconscious, together with his wife, their three children, and three other housemates. Upon investigation by the medical team, it was found that the portable generator was placed inside their house, after experiencing a strong typhoon a day prior. Three family members were already dead, but the remaining unconscious members were brought to various hospitals for further management. Three of the members were brought to our emergency room, and the index patient was referred to different specialties, including the neurology service. Upon examination, the patient had normal vital signs and was hooked to 10 L per minute of oxygen via a face mask for oxygen support along with adequate hydration. The patient was encephalopathic and was seen drowsy with unsustained eye opening, with incomprehensible speech, and unable to follow commands. No lateralizing signs of weakness, numbness, or pathologic reflexes were seen.

Complete blood count (shown in Table 1) showed increased white blood cell count with neutrophilic shift.

**Table 1:** Complete blood count

	Result	Reference
Hemoglobin	16.9	13.5-18.0 g/dL
Hematocrit	48	42%-54%
Red blood cell (RBC)	5.6	4.7-6.0 × 10^12^/L
Mean corpuscular hemoglobin concentration (MCHC)	35	32-36 g/dL
Mean corpuscular hemoglobin (MCH)	30	27-31 pg
Mean corpuscular volume (MCV)	86	78-100 fL
Red cell distribution width (RDW)	12.6	11.5%-15.0%
White blood cell count (WBC)	16.82	5.0-10.0 × 10^9^/L
Neutrophils	85	37%-72%
Lymphocytes	6	20%-50%
Monocytes	9	2%-9%
Eosinophils	0	0%-4%
Basophils	0	0%-1%
Platelet	433	150-440 × 10^9^/L

Upon admission, the patient initially had signs of hepatic injury as evidenced by elevated liver enzymes. Moreover, he had an increasing trend of creatinine as high as 576.3 umol/L (shown in Table 2), but this eventually decreased thereafter.

**Table 2:** Serial blood examination during admission

	Reference	First Hospital Day	Second Hospital Day	Third Hospital Day	Fourth Hospital Day	Fifth Hospital Day	Sixth Hospital Day
Creatinine	64-104 umol/L	198.3	356.1	576.30	552.80	449.30	340.70
Aspartate transferase (AST)	5-34 U/L	1,135	897	357	157	Not extracted	Not extracted
Alanine transferase (ALT)	5-55 U/L	122	158	97	61	Not extracted	Not extracted

Apart from the increased creatinine levels, the patient also presented with muscle tenderness and slightly darker urine output, to which the attending physicians entertained rhabdomyolysis. Table 3 shows increased urine red blood cell count and slight proteinuria. Moreover, muscle enzymes were also markedly elevated at first but had decreasing trends as aggressive hydration continued (shown in Table 4).

**Table 3:** Urinalysis RBC, red blood cell; WBC, white blood cell; HPF, high power field

Urinalysis	Result	Reference
Color	Yellow	Yellow
Appearance	Slightly hazy	Clear
Specific gravity	1.025	1.005-1.025
pH	5.5	4.5-8
Sugar	Trace	Negative
Ketones	Negative	Negative
Blood	+3	0
Nitrite	Negative	Negative
Protein	+2	0
Bilirubin	Negative	Negative
Urobilinogen	Negative	Negative
Leucocytes	Trace	Negative
Urine RBC	24	0-2/HPF
Urine WBC	5	0-2/HPF
Urine epithelial cells	0	0-10/HPF
Bacteria	2	0

**Table 4:** Muscle enzymes

	Reference	First Hospital Day	Second Hospital Day	Third Hospital Day	Fourth Hospital Day	Fifth Hospital Day	Sixth Hospital Day
Total creatine kinase (CK)	30-200 U/L	14,089	4,267	6,708	4,187	3,127	1,830
CK MM (skeletal muscle)	27-175 U/L	13,519	3,916	6,499	4,049	3,006	1,762
CK MB (heart)	3-25 U/L	570	351	209	138	121	68

Due to the unavailability of a pulse carbon monoxide oximetry, a serial determination of blood gas was employed (shown in Table 5). An increasing trend of fraction percentage of carboxyhemoglobin was seen but stabilized after the fourth hospital day. Apart from the carboxyhemoglobin level, the patient’s metabolic acidosis also resolved eventually. During this time, the patient’s neurological status improved significantly as he had sustained wakefulness with correct and consistent responses.

**Table 5:** Serial arterial blood gas determination pCO_2_, partial pressure of carbon dioxide; HCO_3_, bicarbonate; pO_2_, partial pressure of oxygen

Arterial Blood Gas	First Hospital Day	Second Hospital Day	Third Hospital Day	Fourth Hospital Day	Fifth Hospital Day	Sixth Hospital Day
pH (7.35-7.45)	7.312	7.284	7.338	7.453	7.406	7.472
pCO_2_ (35-45 mmHg)	36.5	35.3	37	35	40.4	33.3
HCO_3_ (22-26 mEq/L)	18.7	17.2	20	25.2	24.9	25.5
pO_2_ (80-100 mmHg)	312	287	246	75.5	75.4	87.2
Saturated O_2_ (%) (94%-100%)	97.7	97.8	98.1	95.1	94.6	96.6
Fraction of inspired oxygen (FiO_2_) (%)	60	40	60	40	40	40
Fraction of oxygenated hemoglobin (FO_2_Hb) (%)	93.6	94	94.4	91	90.6	92.1
Fraction of carboxyhemoglobin (FCOHb) (%)	0.2	0.3	0.4	0.9	0.8	0.7
Fraction of methemoglobin (FMetHb) (%)	4	3.6	3.4	3.4	3.4	3.2

**Figure 1:**
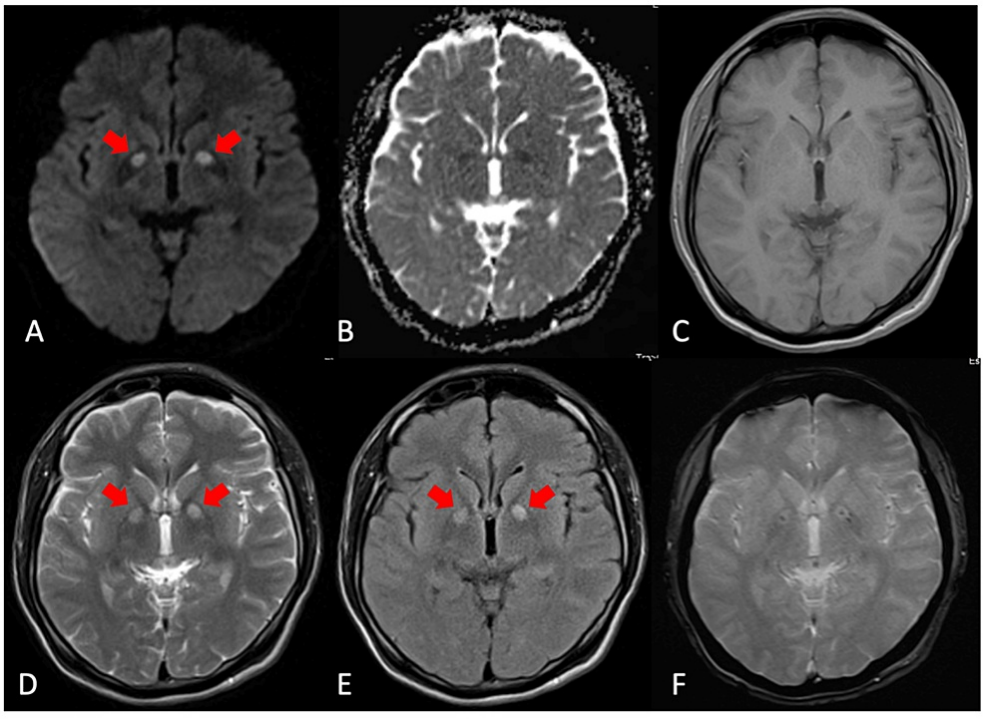
MRI axial, cross-sectional view A) Diffusion-weighted imaging (DWI), B) apparent diffusion coefficient (ADC) map, C) T1-weighted, D) T2-weighted, E) T2 fluid-attenuated inversion recovery (FLAIR), and F) gradient echo (GRE) The red arrows pointing to lesions situated around the bilateral globus pallidus MRI: magnetic resonance imaging

He also underwent cranial magnetic resonance imaging (MRI) (seen in Figure 1), which showed restricted diffusion in the bilateral globus pallidus area on diffusion-weighted imaging (DWI) sequence with associated dropout signal in apparent diffusion coefficient (ADC) map. Moreover, there is a low signal intensity in the T1-weighted image with hyperintense signals on T2-weighted and fluid-attenuated inversion recovery (FLAIR) images. On gradient echo (GRE) imaging, tiny foci of increased susceptibility are noted, which may represent microhemorrhages. All the findings represented changes seen in post-hypoxic encephalopathy, consistent with the patient’s exposure to carbon monoxide.

The patient was eventually started physical rehabilitation for a continuous and steady recovery. Moreover, he was referred to a psychiatry service for trauma-specialized psychotherapy. He was also started with an antidepressant prior to discharge. The two other family members admitted to our institution were also discharged well and stable.

## Discussion

Carbon monoxide is a colorless, nonirritating gas, which is a byproduct of combustion. Fire-related smoke inhalation is responsible for the majority of CO poisoning cases. However, it is also seen in fuel-burning devices, gasoline-powered generators, and motor vehicles operating in poorly ventilated spaces [3,6].

As the carbon monoxide gets inhaled, it binds to the iron moiety of heme with significant affinity compared to oxygen. This then results in decreased oxygen delivery to tissues, leading to subsequent damage [7]. A wide range of symptoms, from nonspecific constitutional symptoms such as headache, drowsiness, and fatigue to altered mental status leading to coma, can be seen. However, it all depends on the fraction inspired percentage of carbon monoxide, the patient’s baseline comorbidities, and the duration of exposure to the toxin [8].

Studies have also demonstrated carbon monoxide intoxication producing delayed neurological sequelae (DNS), which appear around 2-40 days after a lucid interval post intoxication, followed by recurrent intermittent neuropsychiatric symptoms [6,9]. The MRI findings of such showed lesions in the basal ganglia, white matter, or globus pallidus [9], which were present in our index patient.

Rhabdomyolysis as a complication of carbon monoxide poisoning has been reported in the literature. Muscle necrosis secondary to CO poisoning leads to the release of myoglobin from the skeletal muscle, which in turn causes acute kidney injury [10]. Our patient had muscle tenderness with increased muscle enzymes. Adequate hydration was given along with continuous close surveillance reversing the initial acute kidney injury.

Hyperbaric oxygen therapy has been regarded as the initial therapy for acute carbon monoxide poisoning. However, this alone as a standard therapy is still unclear and remains controversial [6,7,9]. Also, this therapy still has undetermined effects on the carbon monoxide-associated delayed neurological sequelae [9].

## Conclusions

This case report highlights a presentation of unintentional carbon monoxide poisoning in a Filipino household. Furthermore, this emphasizes the importance of history and high clinical suspicion to aid in the early recognition of an exposure to carbon monoxide or to any chemicals, which can lead to prompt intervention and treatment, hence avoiding permanent neurological injury.
